# Expression of Concern: Risk factors of progression to endometrial cancer in women with endometrial hyperplasia: A retrospective cohort study

**DOI:** 10.1371/journal.pone.0347720

**Published:** 2026-04-22

**Authors:** The PLOS One Editors

The *PLOS One* Editors issue this Expression of Concern because this article [1] was identified as one of a series of submissions for which we have concerns about the peer review process. Readers are advised to interpret the article [1] with caution.
